# Determining the geographic origin of invasive populations of the mealybug *Planococcus ficus* based on molecular genetic analysis

**DOI:** 10.1371/journal.pone.0193852

**Published:** 2018-03-22

**Authors:** Kent M. Daane, Mathew C. Middleton, René F. H. Sforza, Nicholas Kamps-Hughes, Gillian W. Watson, Rodrigo P. P. Almeida, Margarita C. G. Correa, Doug A. Downie, Vaughn M. Walton

**Affiliations:** 1 Department of Environmental Science, Policy and Management, University of California, Berkeley, California; 2 USDA-ARS- European Biological Control Laboratory, Campus International de Baillarguet CS90013 Montferrier-sur-Lez, St-Gély du Fesc, France; 3 California Department of Food & Agriculture, Plant Pest Diagnostic Center, Sacramento CA, United States of America; 4 Université Côte d'Azur, INRA, CNRS, Institut Sophia Agrobiotech, Sophia Antipolis, France; 5 Department of Pesticide Regulation, Pest Management and Licensing, Sacramento, CA; 6 Department of Horticulture, Oregon State University, Corvallis, Oregon, United States of America; National Cheng Kung University, TAIWAN

## Abstract

Determining the most likely source of an invasive pest species might help to improve their management by establishing efficient quarantine measures and heading the search of efficient biological control agents. *Planococcus ficus* is an invasive mealybug pest of vineyards in Argentina, California, Mexico, Peru and South Africa. This mealybug pest had a previously known geographic distribution spanning southern Europe, the Middle East, and parts of northern Africa. In North America, *Pl*. *ficus* was first discovered in the early 1990s and soon thereafter in Mexico. To determine the origin of invasive populations in North America, *Pl*. *ficus* from California and Mexico were compared with material throughout its presumptive native range in the Mediterranean region, as well as material collected from an older invasion in South Africa and recently invaded Argentina. From each sample location, genomic DNA was sequenced for the nuclear internal transcribed spacer one (ITS1) and the mitochondrial cytochrome c. oxidase one (CO1). Phylogenetic analyses of CO1, ITS1 and concatenated CO1 and ITS1 data-sets using Bayesian and neighbor-joining analysis support two major divisions: a European grouping (Europe, Tunisia, Turkey) and a Middle Eastern grouping (Israel and Egypt). The invasive populations in Argentina and South Africa align with the European group and the invasive populations in North America align with the Middle Eastern group, with one Israel sample aligning closely with the North American clade, suggesting that Israel was the origin of those populations.

## Introduction

Invasive arthropods have hampered the adoption of sustainable vineyard management practices in many of the world’s grape regions, and mealybugs (Hemiptera: Pseudococcidae) are one of the more problematic and invasive vineyard pests [1]. Several mealybug species have risen to the level of economically damaging invasive species, including obscure mealybug (*Pseudococcus viburni* (Signoret)), long-tailed mealybug (*Ps*. *longispinus* (Targioni Tozzetti)), citrophilus mealybug (*Ps*. *calceolariae* (Maskell)), vine mealybug (*Planococcus ficus* (Signoret)), citrus mealybug (*Pl*. *citri* (Risso)), and pink hibiscus mealybug (*Maconellicoccus hirsutus* (Green)) [2–4]. As these mealybugs feed, they excrete carbohydrate-rich honeydew, which can accumulate on the leaves and grape clusters and act as a substrate for sooty mold growth [5]. For table grape growers, the presence of any mealybugs, honeydew or sooty molds in the grape cluster reduces marketability [6]. However, for the world’s wine grape growers, the transmission of viruses vectored by mealybugs, rather than just mealybug feeding or contamination, is their primary concern [7–10].

Quarantine regulations and national programs are commonly instituted to prevent the movement of pests and diseases between grape-growing regions; this includes the use of pest- and pathogen-free plant material [11, 12]. In many countries, there are procedures in place to prevent the movement of mealybugs and other grape pests; for example, hot water dipping and other procedures to clean nursery stock [13, 14]. Still, there continues to be concern about regional and international movement of pest species and there is no standard global procedure used within or between all regions [1]. In North America, *Pseudococcus maritimus* Ehrhorn is believed to be the only native mealybug pest in vineyards and is the primary mealybug found in most US and Canadian vineyards [3, 4, 15]. While the invasive *Ps*. *viburni* and *Ps*. *longispinus* have long been found in North America, they are commonly only vineyard pests in coastal regions of California [16]. Therefore, *Pl*. *ficus* is the most damaging invasive vineyard mealybug in North America, where it was first identified in California’s Coachella Valley in the early 1990s and soon thereafter in Mexico [17]. The reported distribution of *Pl*. *ficus* includes its presumptive native range of southern Europe, the Middle East, and parts of northern Africa [4], and invaded regions not only California and Mexico, but South Africa [18], Argentina [19] and Peru. Currently *Pl*. *ficus* is not reported from many important grape-growing regions of North America (Canada and the US states of Washington, Oregon, New York, Michigan, Pennsylvania), South America (Chile, Brazil and Uruguay), Australia, New Zealand and China. All vineyard regions may be at risk as *Pl*. *ficus* appears capable of surviving across a wide geographic range, from desert table grapes to cool coastal wine grapes [20], with 3–10 generations per year, depending on temperature. For this reason, its continued spread in North America and other global grape regions remains a concern. Understanding the origin of *Pl*. *ficus* populations in North America may help prevent its movement to other grape-growing regions where it is currently not found, or aid in the search for natural enemies that evolved with the invasive pest population and may be best suited for classical biological control.

One factor hampering the rapid initiation of eradication or suppression programs against invasive mealybug populations is their proper identification. For example, it was particularly difficult to separate *Ps*. *maritimus* and *Ps*. *viburni* until detailed taxonomic descriptions of these closely related species were provided [21]. Separation of *Pl*. *ficus* and *Pl*. *citri* based on morphology is similarly difficult and can be made only through careful slide preparation to discern slight differences in multilocular pores and tubular ducts on adult females, involving the use of a discriminant function score based on six characters [22]. Identification of closely related mealybug species has greatly improved with the development of molecular techniques [23–27]. The purpose of this study is to use molecular tools to determine the geographic origin of invasive populations of *Pl*. *ficus* occurring in California and Mexico. We studied the population genetic structure of *Pl*. *ficus* from Europe, Africa, the Middle East, Argentina, California and Mexico to, first, characterize and determine the origin of the California populations, second, better detect and prevent future movement of this pest in North America and other grape-growing regions and, third, support future studies on the biological control of this pest.

## Materials and methods

### Specimen collections

From 2004 to 2008, fresh specimens from 52 putative *Pl*. *ficus* populations were collected in vineyards from North America (California and Mexico) and South America (Argentina), and vineyards and fig trees in Europe (France, Greece (Crete only), Italy, Portugal, and Spain), Africa (Egypt, Tunisia, and South Africa) and the Middle East (Israel and Turkey) (S1 Table) and preserved immediately in 70–100% ethanol. *Planococcus citri*, a close relative to *Pl*. *ficus* [28], was also collected from citrus in Greece, Tunisia and California in a similar manner to serve as a phylogenetic outgroup (S1 Table). All samples were sent to the University of California, Berkeley where they were prepared for DNA extraction.

When additional suitable material was available, museum-quality slide mounts were made. One to four specimens from each of 13 mealybug populations were prepared as archival-quality slide mounts, for identification based on morphological characters. This involved dissection in 10% KOH and removal of the body contents; soaking in 80% alcohol acidified with a small amount of 10% hydrochloric acid; staining in dilute aqueous acid Fuchsin; de-waxing in Histoclear: phenol (3:1 by volume); clearing in anhydrous clove oil; and mounting the specimens in Canada balsam, using the method described in Watson & Kubiriba [29] and Sirisena et al. [30]. The specimens were examined under a Zeiss compound microscope in phase contrast illumination, at 25–400× magnifications. They were identified using the key to adult female *Planococcus* in Cox [22]

### Genetic data collection

From each sample location, genomic DNA was extracted from at least one whole insect with the DNeasy tissue kit (Qiagen, Inc., Valencia, California, USA). Data were generated for the 497 bp of the nuclear internal transcribed spacer one (ITS1) intron using primers CAS18sF1 5'-TAC-ACA-CCG-CCC-GTC-GCT-ACT-A-3' and 5p8sB2 5'-AAC-CTG-CGG-ATT-ACA-CGA-CGA-3' [31]. An initial denaturing step at 94°C for 4 min was followed by 35 cycles of 20 s at 95°C, 1 min at 58°C, and 1 min at 72°C; with a final extension of 2 min at 72°C. In addition, data were also generated for a 706 bp segment of the mitochondrial cytochrome c. oxidase one (CO1) gene initially using primers Pat 5' TCC- AAT-GCA-CTA-ATC-CAT-ATT-A 3' and Jerry 5' CAA-CAT-TTA-TTT-TGA-TTT TTT-GG 3' [32]. Subsequently, the *Pl*. *ficus* specific primers, forward GK 5’-CAG-GAT-TTG-GTG-CTA-TAT-CTC-3’ and reverse GF 5’-TAG-GAG-AAT-TAT-TTA-ATC-AT-3’, were developed to improve PCR results.

For both sets of CO1 primers, an initial denaturing step at 92°C for 2 min was followed by 35 cycles of 1 min 30 sec at 92°C, 1 min 30 sec at 47°C, and 2 min 30 sec at 72°C; with a final extension of 7 min at 72°C. All amplifications were performed in a Biometra T- personal thermal cycler (Biometra Göettingen, Germany) using Taq PCR Master Mix Kit (Qiagen) with a MgCl_2_ concentration of 1.5 mM and 0.25 μM of each primer. For each reaction, 1 μl of genomic DNA (unknown concentration) was used for a total reaction volume of 12.5 μl. PCR products were visualized after electrophoresis on a 1.2% agarose gel, stained with ethidium bromide and cleaned using QIAquick PCR Purification Kit (Qiagen). Purified PCR product was submitted to the University of California, Berkeley DNA sequencing facility for direct sequencing of both strands using the ABI Big Dye V3.1 terminator sequencing reaction kit (Perkin-Elmer/ABI, Weiterstadt, Germany) on an ABI 3707xl DNA Analyzer (Perkin-Elmer) with POP 7 and a 50 cm array. DNA sequences were analyzed and aligned in SeqMan 2 version 5.07 (DNASTAR, Madison, Wisconsin, USA) and multiple alignments were done in MEGA 5 [33] on the CO1 and concatenated CO1, ITS1 sequences.

### Genetic diversity and phylogeographic analysis

Genetic data were organized into haplotypes sampled at each country for the CO1, ITS1 and concatenated CO1 and ITS1 data sets. DnaSP v. 5.10.01 [34] was used to examine polymorphism among and within the sample populations by excluding sites with gaps and missing data.

Phylogenetic analyses were then completed on both data sets using Bayesian analysis. For model-based analysis, the model of sequence evolution best fitting the data were determined by testing 24 models using a likelihood procedure implemented in MEGA5 [33]. For CO1, GTR+G+I [35] was the best model while the ITS1 data fit the K2 parameter model [36]. A mixed model approach was used for the concatenated data set. Bayesian analysis consisted of two chains running for 1,500,000 generations at a temperature of 0.2°C. Chains were sampled every 1000 generations with a burn in of 25000 [37]. Analyses were considered convergent when the average standard deviation of split frequencies was <0.01. Additionally, the genealogical and geographical relationship between haplotypes were analyzed using the median-joining algorithm as implemented in PopArt [38].

## Results

### Morphological identification

All specimens collected from vineyards and fig trees were identified as *Pl*. *ficus*, based on the available keys and except for two *Pl*. *citri* populations sampled as an outlier group. Archival-quality voucher slide mounts of 1–4 specimens from each of 13 sampled populations were deposited in the California State Collection of Arthropods at the Plant Pest Diagnostic Center, Sacramento, California, USA. The collection data for these samples are given in S1 Table.

### Genetic diversity and phylogeography

The CO1 gene region was successfully amplified and direct sequenced using both pairs of CO1 primers for 93 individuals. The ITS1 gene, however, was more problematic during PCR and only some samples were successfully sequenced. Data are available on GenBank® for both the CO1 and ITS1 respectively (accession numbers are listed in S1 Table). The CO1 consisted of 37 haplotypes with a mean of 27.9 nucleotide substitutions over 706 bp for the most divergent groups (Table 1) and the average number of pairwise nucleotide divergences (κ) was 12.8 bp. In comparison, the ITS1 was less variable than the CO1, and produced only 9 haplotypes with a mean of 4.0 nucleotide substitutions over 497 bp for the most divergent groups (Table 1) and the average number of pairwise nucleotide divergences (κ) was 1.8 bp.

**Table 1:** DNA sequence variation.

CO1/ ITS1	Country where the mealybug population was sampled												
	Argentina	Egypt	France	Greece	Israel	Italy	Mexico	Portugal	So. Africa	Spain	Tunisia	Turkey	USA
**Argentina (3/3)**	0/1.3												
**Egypt (3/3)**	23/4.0	0/0.667											
**France (10/8)**	3.4/1.3	25.2/2.6	1.9/0.0										
**Greece (5/3)**	8.0/2.3	25.6/1.6	9.0/1.0	6.6/0.0									
**Israel (4/3)**	21.5/2.5	6.5/3.6	23.8/3.0	24.2/2.0	4/2.6								
**Italy (9/4)**	6.3/2.3	26.1/1.6	6.3/1.0	6.7/0.0	24.7/2.0	4.5/0.0							
**Mexico (6/6)**	24.0/3.2	9.0/3.0	26.6/2.3	26.4/1.3	7.0/2.2	27.4/1.3	0/1.3						
**Portugal (11/2)**	2.2/2.3	24.7/2.6	1.8/1.0	7.7/0.5	23.2/2.5	5.1/0.5	25.7/1.8	0.8/2.0					
**So. Africa (2/2)**	3.5/1.3	26.5/2.6	2.9/0.0	8.7/1.0	25.0/3.0	6.5/1.0	27.5/2.3	1.9/1.0	1.0/0.0				
**Spain (12/4)**	2.3/1.8	24.5/2.6	2.2/0.5	8.0/1.0	23.0/3.0	5.6/1.0	25.5/2.3	1.1/0.7	2.3/0.5	1.4/1.0			
**Tunisia (13/3)**	8.3/1.6	26.8/3.6	9.5/3.0	6.1/2.0	25.4/1.3	6.2/2.0	27.8/2.6	8.3/3.0	9.8/3.0	8.6/3.0	3.1/0.0		
**Turkey (7/5)**	7.2/2.3	25.7/1.6	9.2/1.0	7.4/0.0	24.9/2.0	6.8/0.0	27.0/1.3	7.9/0.5	9.3/1.0	8.1/1.0	7.5/2.0	3.6/0.0	
**USA (8/6)**	24.1/3.0	9.1/3.6	26.7/3.0	26.5/2.0	7.1/2.0	27.5/2.0	.125/2.0	25.8/2.5	27.6/3.0	25.6/3.0	27.9/2.0	27.1/2.0	0.2/2.0

DNA sequence variation in 706 bp of the CO1 and 497 bp of the ITS1 gene regions from *Planococcus ficus* (Hem.: Pseudococcidae) sampled populations; for each row and column, the average number of pairwise difference, within (diagonal element) and between 13 population groups (below diagonal) is shown, with the CO1 / ITS1 gene regions on the left and right, respectively.

**Fig 1:**
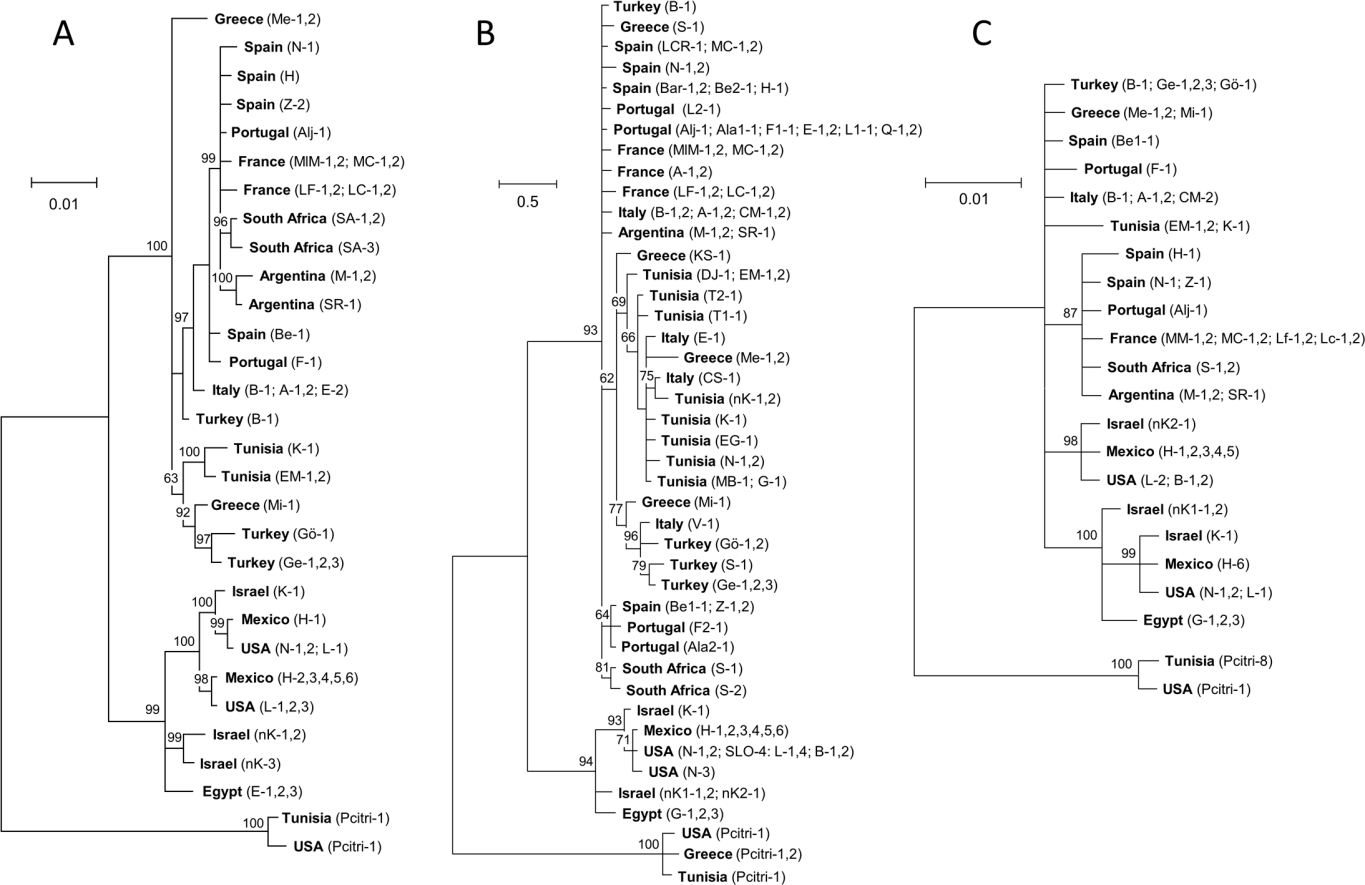
(A) Consensus trees for populations of *Planococcus ficus* (and the out-group *Pl*. *citri*) derived from Bayesian analysis, with the numbers at nodes showing posterior probabilities, using (A) a concatenated CO1 and ITS1 data set, (B) the COI data set, and (C) the ITS1 data set; the insect populations were collected in Europe, Africa, the Middle East, Argentina, California and Mexico and presented grouped by the nearest city and the isolate tested (codes are in Supplemental Table 1).

With the concatenated CO1 and ITS1 data set, Bayesian analysis supports two major divisions of mealybugs: a Mediterranean group (France, Greece, Italy, Portugal, Spain, Tunisia and Turkey) with invasive populations in South Africa and Argentina derived from this grouping, and a Middle Eastern group (Israel and Egypt) with invasive populations in California and Mexico derived from this grouping (Fig 1A). There was weaker support for further separation of the Mediterranean group into a European subgroup (Portugal, Spain, France, and Italy) with invasive populations in South Africa and Argentina derived from this grouping, and a Mediterranean subgroup (Greece, Turkey and Tunisia). However, some populations from Italy (B-1; A-1,2; and E-2), Greece (Me-1,2) and Turkey (B-1) are not strongly separated between the ‘European’ and ‘Mediterranean’ subgroupings (Fig 1A).

The concatenated data set had fewer overall successfully sequenced samples with both CO1 and ITS1; nevertheless, the CO1-only and ITS1-only data sets provided largely similar groupings, with clear support for Mediterranean and Middle Eastern groups (Fig 1B). With the larger CO1-only data set, there was only weak support for further separation of Europe (Portugal, Spain, France, and Italy) from the more southern Mediterranean regions (Greece, Turkey and Tunisia). Moreover, some populations from Greece (S-1) and Tukey (B-1) had no separation from the European subgroup populations in Italy, from Volterra (V-1) in central Italy, Castelsardo (CS-1) in Sardinia, and Erice (E-1) in Sicily that were not separated from most populations collected in Greece, Turkey and Tunisia (Fig 1B). As with the concatenated data set, invasive populations in South Africa and Argentina were in the Mediterranean grouping and invasive populations in North America were with the Middle Eastern grouping.

**Fig 2:**
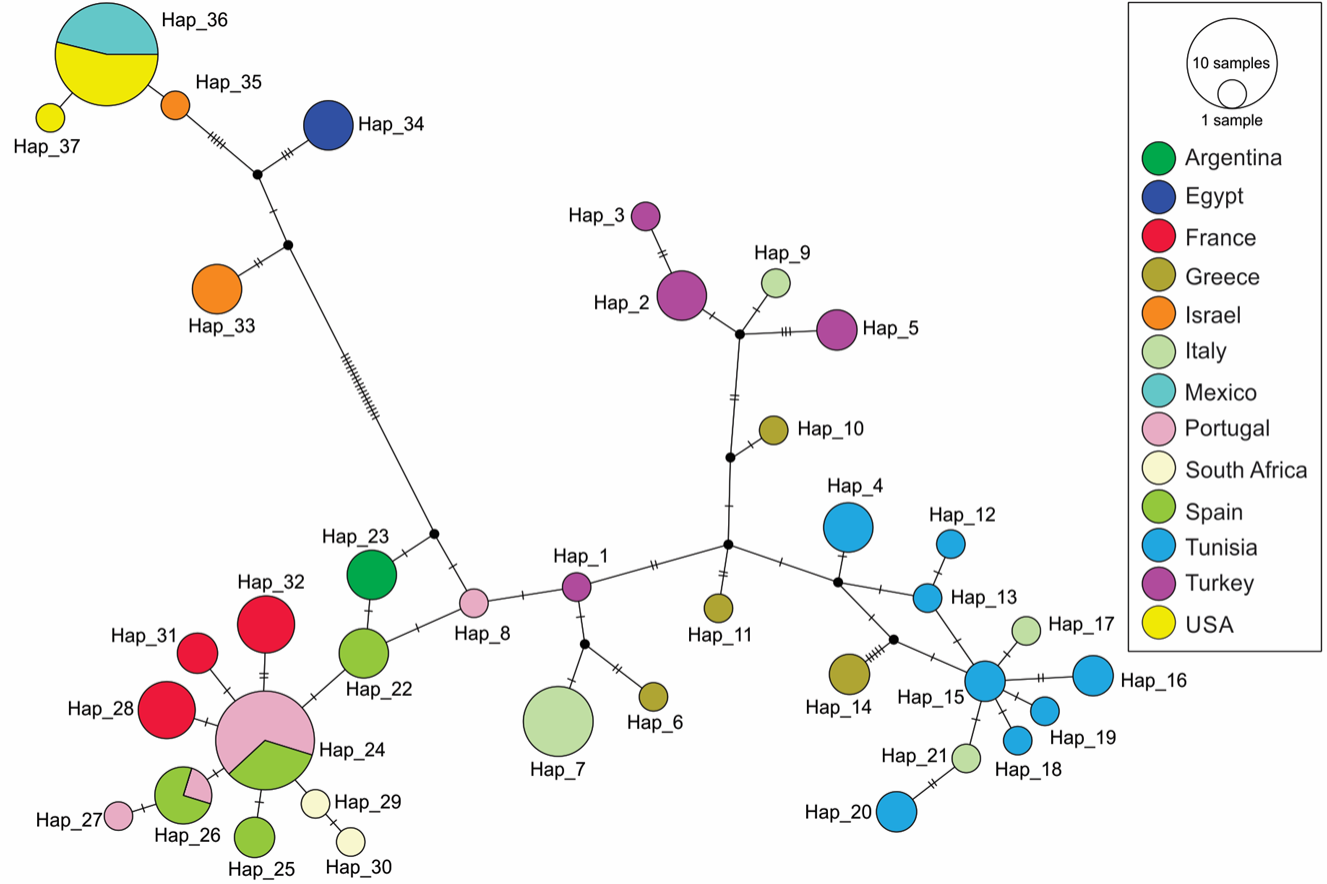
Neighbor-joining network of CO1 data. Haplotype network for the CO1 region for populations of *Planococcus ficus* collected in Europe, Africa, the Middle East, Argentina, California and Mexico. Each circle represents a different haplotype and the sizes of circles correspond to the number of individuals sharing this haplotype. Colors indicate sampling country. The crossbeam on the connecting lines between haplotypes represents a substitution. Black dots symbolize hypothetic haplotypes not sampled in the data set.

**Fig 3:**
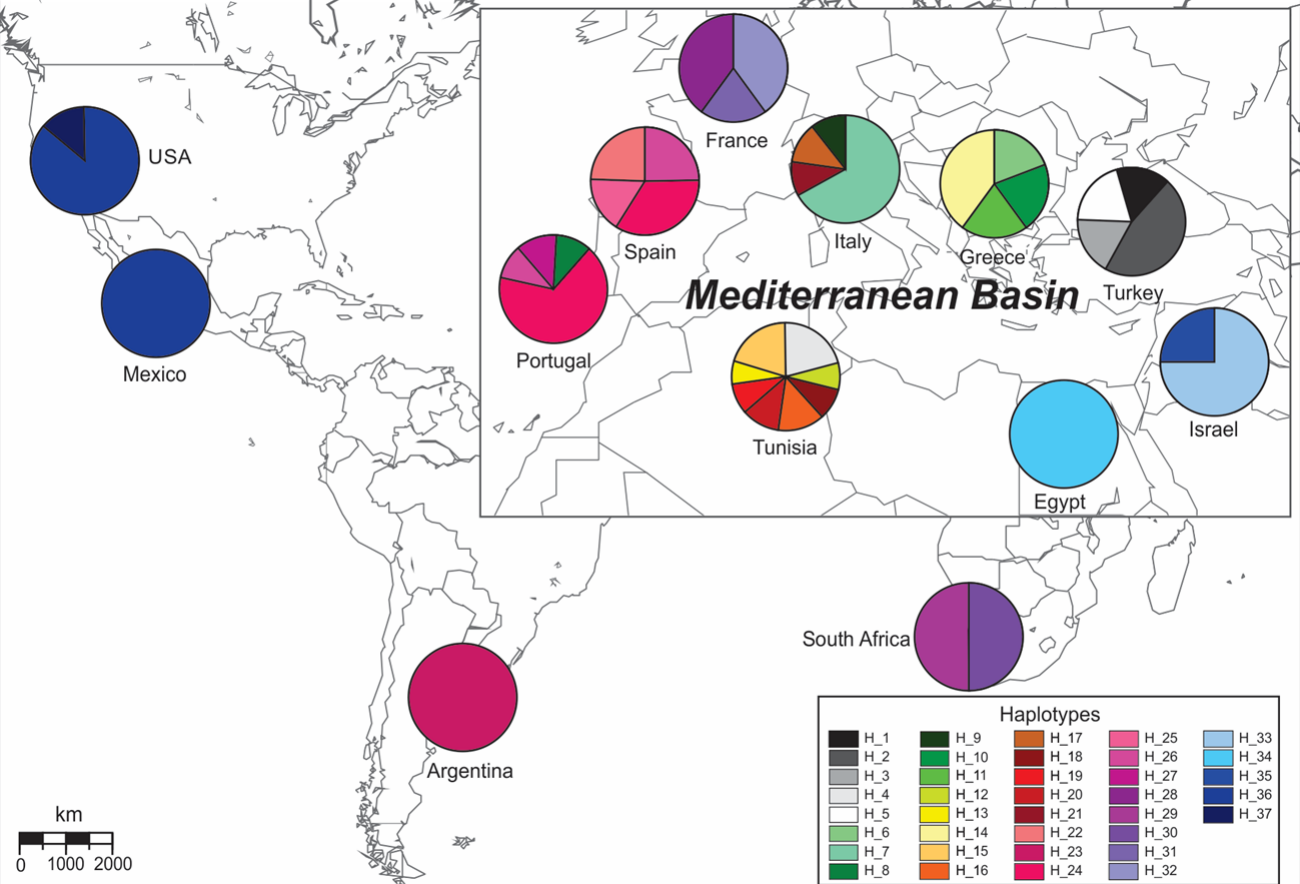
Geographical distribution of the CO1 haplotypes for populations of *Planococcus ficus* collected in Europe, Africa, the Middle East, Argentina, California and Mexico.

The CO1 Neighbor-Joining network also supported Mediterranean and Middle Eastern groupings for the collected *Pl*. *ficus* populations (Fig 2). Whereas haplotypes 1–32 from Europe, Tunisia, Argentina and South African populations grouped together in the base of the network, haplotypes 33–37 from the Middle East and North America constitute a second group separated by 18 mutations from the previous one. haplotypes 15 and 24 appeared to be the most ancestral ones sampled in this study, as they give direct origin to 6 and 7 other haplotypes, respectively. From the 37 CO1 haplotypes, 34 corresponded to private haplotypes for each sampling site (country or city sampled), whereas haplotypes 24 and 26 were shared by Spain and Portugal and haplotype 36 by California and Mexico (Fig 3).

## Discussion

Both Bayesian analysis and CO1 Neighbor-Joining support two broad groupings of the sampled *Pl*. *ficus* populations. The Mediterranean group includes Greece, France, Italy, Portugal, Spain, Turkey, Tunisia, South Africa and Argentina; the Middle Eastern group consists of populations from Egypt, Israel, and North America (California and Mexico). Bayesian analysis shows some support for additional population structure within the Mediterranean group where ‘western European’ subgroup (France, Italy, Portugal and Spain) and invasive populations in Argentina and South Africa, are separate from a ‘southern Mediterranean’ subgrouping (Greece, Turkey and Tunisia), resulting in a total of three geographic groupings (Fig 1A). Using the CO1-only and ITS1-only data sets in Bayesian analysis there is also support for the Mediterranean and Middle Eastern groups, but much weaker support for any further separation of the Mediterranean populations. For example, some haplotypes from Greece and Turkey align more closely with western European grouping and two haplotypes from Italy align more closely with the southern Mediterranean grouping (Fig 1B).

CO1 Neighbor-Joining analysis similarly mapped a strong separation of Mediterranean and Middle Eastern populations (Fig 2) and showed some divergence of the Mediterranean populations similar to the Bayesian analysis of the concatenated data set. Populations from Italy (haplotypes 7, 9, 17, and 21), Greece (haplotypes 6, 10, 11, and 14) and Turkey (haplotypes 1, 2, 3, and 5) had the greatest divergence within any country (Fig 2). This can be explained, in part, by geographic separation of the collection sites and the ‘island’ isolation of some of the samples. For example, the Italian material in the western European subgrouping (Fig 1B) was collected from southern to northern Italy (Bari, Altamura, Padua, and Castel de Monte), whereas the Italian populations in the southern Mediterranean subgrouping were collected from Sicily and Sardinia, although a population from Volterra, Italy also aligned with this group. Similarly, the western European population from Turkey was from Bursatd, which is to the north of the other Turkish collection sites (Fig 1B, Fig 2, S1 Table).

Phylogeography provides information regarding the movement of *Pl*. *ficus* populations within its native range and to different parts of the world. Within the Mediterranean region, the common ancestral haplotype 24 for western Europe (France, Spain and Portugal) indicates exchange of infested material between these countries (Figs 2 and 3). The same situation was present for Tunisia, Italy and Greece with haplotype 15. Other haplotypes (1–3, 5–7 and 9–11) from Greece, Turkey and Italy did not have a clear haplotype of origin, being located between networks of haplotypes 24 and 15. For the invasive populations, both Argentina (haplotype 23) and South African (haplotypes 29 and 30) *Pl*. *ficus* populations were likely introduced from western Europe, as also suggested by Bayesian analysis of the concatenated data set. This suggests that infested plant material shipped from Europe was the likely source of these invasion events. *Planococcus ficus* from North America likely originated from the Middle Eastern group as one haplotype from Israel (haplotype 35) is most closely related to those from California and Mexico (haplotype 36) being separated by only one substitution. The presence of haplotype 37, the most abundant in California, as the unique one present in Mexico suggest a single invasion event that was shared by both California and Mexico. Indeed, the initial North American discovery of *Pl*. *ficus* was in a vineyard in Coachella Valley, a desert region in southern California. After our molecular work was completed, one of the authors (KMD) was informed that a few years before *Pl*. *ficus* had been identified in North America, a table grape grower near the initial infestation had brought varietal cuttings from Israel, and that this same grower farmed in Mexico near Hermosillo, where the *Pl*. *ficus* infestation was first found in that region. This hearsay evidence is presented only to emphasize the importance of planting with certified nursery material that is known to be free of plant diseases and arthropod pests [11].

Analysis of both pairwise nucleotide divergences (Table 1) and haplotypic diversity (Fig 3) supports *Pl*. *ficus* species divergence. Some groups of morphologically similar mealybugs may contain cryptic species, and molecular phylogenetics provides an invaluable tool to help resolve taxonomic challenges [23, 39]. For example, a taxonomic synopsis of the mealybug genus *Ferrisia* initially recognized eleven species [40]; however, a study of *Ferrisia* on California pistachios, using a combination of molecular and morphological diagnostics lead to the description of a new species–*F*. *gilli* Gullan [41]. Further study of the genus worldwide subsequently identified a total of 23 species [42]. Mealybugs from the genus *Planococcus* are also known to present taxonomic difficulties [43]. Our research supports the need for further study of *Pl*. *ficus* speciation. The CO1 p-distance between these groups is around 4%, higher than typical intraspecific variation (which is usually not higher than 2% [44]). However, this value does not indicate a cryptic species as a large survey of CO1 p-distances for congeneric species pairs typically averages 8–16% [45], placing the 4% value lower than expected. Regardless of this value, it would be unwise to utilize average *P*-values that separate congeneric species pairs from different orders of insects to dismiss a potential cryptic species within *Pl*. *ficus*, because values vary depending on the insect order [45, 46]. Useful information on *P*-values that distinguish species within *Planococcus* can be gathered by comparing data from Rung et al. [43] with our data on *Pl*. *ficus*. The CO1 *P*-distance between the closely related *Pl*. *minor* and *Pl*. *citri* is only 2% [43], whereas the distance between *Pl*. *ficus* and *Pl*. *minor* is 7.1% and between *Pl*. *ficus* and *Pl*. *citri* is 7.5%, scoring near-average values. Consequently, our value of 4% falls within a range ~2.0–7.5% variation that occurs between different *Planococcus* species. Moreover, haplotypic diversity of the sampled *Pl*. *ficus* between the Mediterranean grouping (Europe, Turkey, Tunisia, Argentina and South Africa) with 32 haplotypes and Middle Eastern grouping (Israel, Egypt and North America) with 5 haplotypes was contrasting (Fig 3). The high diversity within the broader Mediterranean group suggests that our samples were representative of some of the genetic diversity of the presumptive native range of *Pl*. *ficus*, whereas further sampling is needed to correctly characterize diversity of the Middle Eastern group. The question of whether the Mediterranean and Middle Eastern *Pl*. *ficus* groups represent cryptic species cannot be determined until a study includes morphological evaluations of *Pl*. *ficus* along with analysis of further molecular markers (i.e. microsatellites). We note that our slide-mounted specimens were identified morphologically as *Pl*. *ficus* using the available taxonomic keys.

It may also be important to understand pest origin and the possible existence of cryptic species for successful execution of classical biological control [47, 48]. One of the more important natural enemies of *Pl*. *ficus* is the parasitoid *Anagyrus pseudococci* (Girault) (Hymenoptera: Encyrtidae). *Anagyrus pseudococci* has been developed as a biological control agent in California to help manage *Pl*. *ficus*-infested vineyards [49]. Improving natural suppression of *Pl*. *ficus* in California vineyards may provide an alternative to, or an improvement of, pesticide use [50]. Recent molecular and morphological work showed the existence of morphotypes of *A*. *pseudococci*, and Triapitsyn et al. [51] designated the name *A*. *pseudococci* to populations from Argentina and Cyprus, while populations from Brazil, Palearctic Asia and North America, and parts of Italy were designated as *Anagyrus* sp. near *pseudococci*. Correct identification of a pest and its natural enemies is essential to successful biological control as it may be important to use natural enemies that can attack the targeted pest, and cryptic species may be morphologically identical based on current taxonomic keys, but recognizable to the parasitoid. *Anagyrus* sp. near *pseudococci* had been introduced into California multiple times to control *Pl*. *citri* [52] and later *Pl*. *ficus* [49]. The data presented in this paper also strongly suggest two different native origins for the *Pl*. *ficus* populations introduced in three distant grape geographic regions, North and South America, and South Africa. An increasing interest should be now devoted to establishing biological control programs in Argentina and South Africa that select source populations of the biological control agents *A*. *pseudococci* and *A*. *s* sp. near *pseudococci* in the Euro-Mediterranean regions.

## Supporting information

S1 Table Sample locations and information collected. Vine mealybug, *Planococcus ficus* (Hem.: Pseudococcidae) collection information showing country and locality (city or region), Global Position Satellite (GPS) coordinates when available, collector and date, whether samples were processed by CO1 or ITS1, if there is a museum deposited slide mount for the location, and GenBank accession number.
(DOCX)

## Abbreviations

ITS1: internal transcribed spacer one
CO1: mitochondrial cytochrome c. oxidase one
Bp: base pair
Min: minute(s)
Sec: second(s)
