# Supplementary material for: Determining the geographic origin of invasive populations of the mealybug Planococcus ficus based on molecular genetic analysis
Source: PLoS One. 2018 Mar 22;13(3):e0193852. doi: 10.1371/journal.pone.0193852 (PMC5863958; doi:10.1371/journal.pone.0193852)
Supplement: S1 Table — Vine mealybug, Planococcus ficus (Hem.: Pseudococcidae) collection information showing country and locality (city or region), Global Position Satellite (GPS) coordinates when available, collector and date, whether samples were processed by CO1 or ITS1, if there is a museum deposited slide mount for the location, and GenBank accession number. (DOCX) [file pone.0193852.s001.docx]

**S1 Table.** **Sample locations and information collected.** Vine mealybug, *Planococcus ficus* (Hem.: Pseudococcidae) collection information showing country and locality (city or region), Global Position Satellite (GPS) coordinates when available, collector and date, whether samples were processed by CO1 or ITS1, if there is a museum deposited slide mount for the location, and GenBank accession number.

| **Country** | **Locality (code)** | **GPS** | **Collector and date** | **CO1** | **ITS1** | **Slide** | **Accession Number** |
| --- | --- | --- | --- | --- | --- | --- | --- |
|  |  |  |  |  |  |  |  |
| Argentina | Mendoza (M) | S 32° 59'16'' W 68° 51'41'' | K Daane, 8-III-2007 | ● |  |  | MF952546-7 |
| Argentina | Mendoza (M) | S 32° 59'16'' W 68° 51'41'' | K Daane, 8-III-2007 |  | ● |  | MF952595-6 |
| Argentina | San Rafael (SR) | S 34° 36'28” W 68° 14'36'' | K Daane, 8-III-2007 | ● |  |  | MF952548 |
| Argentina | San Rafael (SR) | S 34° 36'28” W 68° 14'36'' | K Daane, 8-III-2007 |  | ● |  | MF952601 |
| Egypt | Giza (G) | N 30° 01'25'' E 31° 11'45'' | K Sime, 15-I-2004 | ● |  |  | MF952541-3 |
| Egypt | Giza (G) | N 30° 01'25'' E 31° 11'45'' | K Sime, 15-I-2004 |  | ● |  | MF952600 |
| Egypt | Giza (G) | N 30° 01'25'' E 31° 11'45'' | K Sime, 15-I-2004 |  | ● |  | MF952602 |
| France | Alès (A) | N 44° 13'27'' E 04° 09'48'' | R Sforza, 9-VIII-2005 | ● |  |  | MF952505-6 |
| France | Limoux-Cimetière (LC) | N 43° 07'53'' E 03° 05'00'' | R Sforza, 5-VII-2005 | ● |  |  | MF952511-2 |
| France | Limoux-Cimetière (LC) | N 43° 07'53'' E 03° 05'00'' | R Sforza, 5-VII-2005 |  | ● |  | MF952573-4 |
| France | Limoux-Flandry (LF) | N 43° 07'53'' E 03° 05'00'' | R Sforza, 5-VII-2005 | ● |  |  | MF952509-10 |
| France | Limoux-Flandry (LF) | N 43° 07'53'' E 03° 05'00'' | R Sforza, 5-VII-2005 |  | ● |  | MF952571-2 |
| France | Massif de la Clape (MC) | N 43° 07'53'' E 03° 05'00'' | R Sforza, 31-VIII-2005 | ● |  | ● | MF952507-8 |
| France | Massif de la Clape (MC) | N 43° 07'53'' E 03° 05'00'' | R Sforza, 31-VIII-2005 |  | ● |  | MF952569-70 |
| France | Mur.-lès-Montpellier (MM) | N 43° 35'49'' E 03° 44'41'' | R Sforza, 4-VIII-2005 | ● | ● |  | MF952503-4 |
| France | Mur.-lès-Montpellier (MM) | N 43° 35'49'' E 03° 44'41'' | R Sforza, 4-VIII-2005 |  | ● |  | MF952567-8 |
| Greece | Kato Simi (KS) | N 35° 09'01'' E 25° 33'20'' | R Sforza, 8-VIII-2005 | ● |  |  | MF952479 |
| Greece | Messara (Me) | N 35° 02'35'' E 24° 52'38'' | R Sforza, 3-X-2005 | ● |  | ● | MF952475-6 |
| Greece | Messara (Me) | N 35° 02'35'' E 24° 52'38'' | R Sforza, 3-X-2005 |  | ● |  | MF952559-60 |
| Greece | Mires (Mi) | N 35° 07'37'' E 24° 59'44'' | R Sforza, 7-IX-2005 | ● |  |  | MF952474 |
| Greece | Mires (Mi) | N 35° 07'37'' E 24° 59'44'' | R Sforza, 7-IX-2005 |  | ● |  | MF952558 |
| Greece | Petrokefali (P) | N 35° 01'56'' E 24° 51'06'' | R Sforza, 7-IX-2005 | ● |  |  | MF952477-8 |
| Greece | Samariá (S) | N 35° 14'00'' E 23° 58'00'' | R Sforza, 5-X-2006 | ● |  | ● | MF952473 |
| Israel | Keshet (K) | N 32° 58'00'' E 35° 48'00'' | Z Mendel, 10-VII-2008 | ● |  |  | MF952522 |
| Israel | Keshet (K) | N 32° 58'00'' E 35° 48'00'' | Z Mendel, 10-VII-2008 |  | ● |  | MF952579 |
| Israel | near Keshet (nK1) | not recorded | K Sime, 25-IV-2008 | ● |  |  | MF952523-4 |
| Israel | near Keshet (nK2) | not recorded | K Sime, 25-IV-2008 | ● |  |  | MF952525 |
| Israel | near Keshet (nK1) | not recorded | K Sime, 25-IV-2008 |  | ● |  | MF952580 |
| Israel | near Keshet (nK2) | not recorded | K Sime, 25-IV-2008 |  | ● |  | MF952581 |
| Italy | Altamura (A) | N 40° 59'56'' E 16° 39'51'' | R Sforza, 19-VI-2005 | ● |  |  | MF952515-6 |
| Italy | Altamura (A) | N 40° 59'56'' E 16° 39'51'' | R Sforza, 19-VI-2005 |  | ● |  | MF952576-7 |
| Italy | Bari (N) | N 41° 05'05'' E 16° 48'00'' | R Sforza, 19-VII-2005 | ● |  |  | MF952513-4 |
| Italy | Bari (N) | N 41° 05'05'' E 16° 48'00'' | R Sforza, 19-VII-2005 |  | ● |  | MF952575 |
| Italy | Castel del Monte (CM) | N 41° 04'47'' E 16° 17'23' | R Sforza, 19-VII-2005 | ● |  |  | MF952518-9 |
| Italy | Castel del Monte (CM) | N 41° 04'47'' E 16° 17'23' | R Sforza, 19-VII-2005 |  | ● |  | MF952578 |
| Italy | Castelsardo-Sardinia (CS) | N 40° 57'00'' E 8° 52'20'' | R Sforza, 8-VII-2007 | ● |  |  | MF952521 |
| Italy | Erice-Sicily (E) | N 38° 02'55'' E 12° 35'03'' | R Sforza, 5-VII-2007 | ● |  |  | MF952520 |
| Italy | Volterra (V) | N 43° 28'07'' E 10° 50'22'' | R Sforza, 30-V-2007 | ● |  |  | MF952517 |
| Mexico | Hermosillo-Sonora (H) | N 29° 05'21'' W 110° 57'38'' | A Castillo, 12-V-2008 | ● |  |  | MF952526-31 |
| Mexico | Hermosillo-Sonora (H) | N 29° 05'21'' W 110° 57'38'' | A Castillo, 12-V-2008 |  | ● |  | MF952582-7 |
| Portugal | Alandroal (Ala1) | N 38° 41'47'' E 07° 24'20'' | R Sforza, 8-VII-2005 | ● |  |  | MF952501 |
| Portugal | Alandroal (Ala2) | N 38° 41'47'' E 07° 24'20'' | R Sforza, 8-VII-2005 | ● |  |  | MF952502 |
| Portugal | Aljezur (Alj) | N 37° 19'08'' E 08° 47'51'' | R Sforza, 6-VII-2005 | ● |  |  | MF952494 |
| Portugal | Aljezur (Alj) | N 37° 19'08'' E 08° 47'51'' | R Sforza, 6-VII-2005 |  | ● |  | MF952565 |
| Portugal | Estay (E) | N 37° 05'01'' E 07° 54'11'' | R Sforza, 5-VII-2007 | ● |  |  | MF952497-8 |
| Portugal | Faro (F1) | N 37° 03'44'' E 07° 55'22'' | R Sforza, 7-VII-2005 | ● |  |  | MF952495 |
| Portugal | Faro (F2) | N 37° 03'44'' E 07° 55'22'' | R Sforza, 7-VII-2005 | ● |  |  | MF952496 |
| Portugal | Faro (F) | N 37° 03'44'' E 07° 55'22'' | R Sforza, 7-VII-2005 |  | ● |  | MF952566 |
| Portugal | Lisboa (L1) | N 38° 38'09'' E 08° 40'62'' | R Sforza, 28-IX-2007 | ● |  |  | MF952492 |
| Portugal | Lisboa (L2) | N 38° 38'09'' E 08° 40'62'' | R Sforza, 28-IX-2007 | ● |  |  | MF952493 |
| Portugal | Quinta da Cegognas (Q) | N 37° 09'20'' E 07° 39'32'' | R Sforza, 7-VII-2005 | ● |  | ● | MF952499-500 |
| South Africa | Stellenbosch (S) | S 33° 55′12″ E 18° 51′36″ | V Walton, 18-XII-2007 | ● |  |  | MF952544-5 |
| South Africa | Stellenbosch (S) | S 33° 55′12″ E 18° 51′36″ | V Walton, 18-XII-2007 |  | ● |  | MF952598-9 |
| Spain | Barcelona (Bar) | N 41° 23'16'' E 02° 10'11'' | R Sforza, 2-VII-2005 | ● |  |  | MF952480-1 |
| Spain | Beas (Be1) | N 38° 15'00'' E 03° 02'52'' | R Sforza, 3-VII-2005 | ● |  |  | MF952486 |
| Spain | Beas (Be1) | N 38° 15'00'' E 03° 02'52'' | R Sforza, 3-VII-2005 |  | ● |  | MF952562 |
| Spain | Beas (Be2) | N 38° 15'00'' E 03° 02'52'' | R Sforza, 3-VII-2005 | ● |  |  | MF952487 |
| Spain | Huelva (H) | N 37° 15′00'' W 06° 57'00′' | R Sforza, 5-VII-2005 | ● |  | ● | MF952488 |
| Spain | Huelva (H) | N 37° 15′00'' W 06° 57'00′' | R Sforza, 5-VII-2005 |  | ● |  | MF952563 |
| Spain | Las Cabezas Rutilas (LCR) | N 37° 43'45" W 7° 05'24" | R Sforza, 5-VII-2005 | ● |  |  | MF952491 |
| Spain | Monforte del Cid (MC) | N 38° 21'39'' E 00° 42'05'' | R Sforza, 2-VII-2005 | ● |  | ● | MF952482-3 |
| Spain | Noveddad (N) | N 38° 24'06'' E 00° 46'21'' | R Sforza, 5-VII-2005 | ● |  |  | MF952484-5 |
| Spain | Noveddad (N) | N 38° 24'06'' E 00° 46'21'' | R Sforza, 5-VII-2005 |  | ● |  | MF952561 |
| Spain | Zalamea la real (Z) | N 37° 41' 00" W 6° 39'00" | R Sforza, 5-VII-2005 | ● |  |  | MF952489-91 |
| Spain | Zalamea la real (Z) | N 37° 41' 00" W 6° 39'00" | R Sforza, 5-VII-2005 |  | ● |  | MF952564 |
| Tunisia | Dar el Joundi (DJ) | N 36° 47'01'' E 10° 38'42'' | R Sforza, 1-VII-2006 | ● |  |  | MF952462 |
| Tunisia | El Guetta (EG) | N 34° 19'18'' E 08° 58'46'' | R Sforza, 28-VIII-2005 | ● |  |  | MF952467 |
| Tunisia | El Mrissa (EM) | N 36° 45'02'' E 10° 33'08'' | R Sforza, 1-VII-2006 | ● |  |  | MF952465-6 |
| Tunisia | El Mrissa (EM) | N 36° 45'02'' E 10° 33'08'' | R Sforza, 1-VII-2006 |  | ● |  | MF952554-5 |
| Tunisia | Gabès (G) | N 33° 28'55'' E 10° 34'01' | R Sforza, 29-VIII-2005 | ● |  |  | MF952471 |
| Tunisia | Korbous (K) | N 36° 48'54'' E 08° 58'46'' | R Sforza, 1-VII-2006 | ● |  |  | MF952468 |
| Tunisia | Korbous (K) | N 36° 48'54'' E 08° 58'46'' | R Sforza, 1-VII-2006 |  | ● |  | MF952556 |
| Tunisia | Menzel Bourguiba (MB) | N 37° 09'30'' E 10° 01'17'' | R Sforza, 27-VIII-2005 | ● |  |  | MF952459 |
| Tunisia | near Korbous (nK) | N 36° 49'41'' E 10° 36'42'' | R Sforza, 1-VII-2006 | ● |  |  | MF952469-70 |
| Tunisia | Nefza (N) | N 37° 01'48'' E 09° 06'03'' | R Sforza, 27-VIII-2005 | ● |  | ● | MF952460-1 |
| Tunisia | Thala (T1) | N 35° 34'39'' E 08° 39'50'' | R Sforza, 28-VIII-2005 | ● |  |  | MF952463 |
| Tunisia | Thala (T2) | N 35° 34'39'' E 08° 39'50'' | R Sforza, 28-VIII-2005 | ● |  |  | MF952464 |
| Turkey | Bursa (B) | N 40° 12'53'' E 28° 43'54'' | R Sforza, 23-VII-2005 | ● |  |  | MF952452 |
| Turkey | Bursa (B) | N 40° 12'53'' E 28° 43'54'' | R Sforza, 23-VII-2005 |  | ● |  | MF952549 |
| Turkey | Gelembre (Ge) | N 39° 11'22'' E 27° 51'09'' | R Sforza, 23-VII-2005 | ● |  | ● | MF952453-5 |
| Turkey | Gelembre (Ge) | N 39° 11'22'' E 27° 51'09'' | R Sforza, 23-VII-2005 |  | ● |  | MF952550-2 |
| Turkey | Gölmarmara (Gö) | N 38° 23'06'' E 28° 29'00'' | R Sforza, 23-VII-2005 | ● |  | ● | MF952456-7 |
| Turkey | Gölmarmara (Gö) | N 38° 23'06'' E 28° 29'00'' | R Sforza, 23-VII-2005 |  | ● |  | MF952553 |
| Turkey | Safranbolu (S) | N 41° 14'41'' E 32° 41'35'' | R Sforza, 29-VII-2005 | ● |  |  | MF952458 |
| USA | Bakersfield (B) | N 35° 35'11'' W 119° 10'04'' | K Daane, 12-VIII-2007 | ● |  | ● | MF952539-40 |
| USA | Bakersfield (B) | N 35° 35'11'' W 119° 10'04'' | K Daane, 12-VIII-2007 |  | ● |  | MF952593-4 |
| USA | Lodi (L) | N 38° 08'08'' W 121° 16'45'' | K Daane, 2-IX-2007 | ● |  | ● | MF952537-8 |
| USA | Lodi (L) | N 38° 08'08'' W 121° 16'45'' | K Daane, 2-IX-2007 |  | ● |  | MF952591-2 |
| USA | Napa (N) | N 38°30'11'' W 122° 28'00'' | K Daane, 16-VIII-2007 | ● |  | ● | MF952532-4 |
| USA | Napa (N) | N 38°30'11'' W 122° 28'00'' | K Daane, 16-VIII-2007 |  | ● |  | MF952588-9 |
| USA | San Luis Obispo (SLO) | N 35° 14'15'' W 120° 38'32'' | M Cooper, 22-IX-2007 | ● |  | ● | MF952535-6 |
| USA | San Luis Obispo (SLO) | N 35° 14'15'' W 120° 38'32'' | M Cooper, 22-IX-2007 |  | ● |  | MF952590 |
